# Antimicrobial resistance and virulence characterization of *Staphylococcus aureus* and coagulase-negative staphylococci from imported beef meat

**DOI:** 10.1186/s12941-017-0210-4

**Published:** 2017-05-10

**Authors:** Kamelia Osman, Avelino Alvarez-Ordóñez, Lorena Ruiz, Jihan Badr, Fatma ElHofy, Khalid S. Al-Maary, Ihab M. I. Moussa, Ashgan M. Hessain, Ahmed Orabi, Alaa Saad, Mohamed Elhadidy

**Affiliations:** 1 Department of Microbiology, Faculty of Veterinary Medicine, Cairo University, Giza, Egypt; 2 Department of Food Hygiene and Technology and Institute of Food Science and Technology, University of León, León, Spain; 3 Department of Nutrition, Bromatology and Food Technology, Universidad Complutense de Madrid, Madrid, Spain; 4 Department of Poultry Diseases, Animal Health Research, Institute, Giza, Egypt; 5 Department of Bacteriology, Immunology and Mycology, Faculty of Veterinary Medicine, Benha University, Moushtohor, Egypt; 6 Department of Botany and Microbiology, College of Science, King Saud University, Riyadh, Kingdom of Saudi Arabia; 7 Department of Health Science, College of Applied Studies and Community Service, King Saud University, Riyadh, Kingdom of Saudi Arabia; 8 Department of Bacteriology, Mycology and Immunology, Faculty of Veterinary Medicine, Mansoura University, Mansoura, 35516 Egypt; 9 Foodborne Pathogens, Scientific Institute of Public Health, Juliette Wytsmanstraat 14, 1050 Brussels, Belgium

**Keywords:** Coagulase-positive staphylococci, Coagulase-negative staphylococci, Antibiotic resistance genes, Imported beef meat

## Abstract

**Background:**

The objectives of this study were to characterize the diversity and magnitude of antimicrobial resistance among *Staphylococcus* species recovered from imported beef meat sold in the Egyptian market and the potential mechanisms underlying the antimicrobial resistance phenotypes including harboring of resistance genes (*mecA*, *cfr*, *gyrA*, *gyrB*, and *grlA*) and biofilm formation.

**Results:**

The resistance gene *mecA* was detected in 50% of methicillin-resistant non-*Staphylococcus aureus* isolates (4/8). Interestingly, our results showed that: (i) resistance genes *mecA*, *gyrA*, *gyrB*, *grlA*, and *cfr* were absent in *Staphylococcus hominis* and *Staphylococcus hemolyticus* isolates, although *S. hominis* was phenotypically resistant to methicillin (MR-non-*S. aureus*) while *S. hemolyticus* was resistant to vancomycin only; (ii) *S. aureus* isolates did not carry the *mecA* gene (100%) and were phenotypically characterized as methicillin- susceptible *S. aureus* (MSS); and (iii) the resistance gene *mecA* was present in one isolate (1/3) of *Staphylococcus lugdunensis* that was phenotypically characterized as methicillin-susceptible non-*S. aureus* (MSNSA).

**Conclusions:**

Our findings highlight the potential risk for consumers, in the absence of actionable risk management information systems, of imported foods and advice a strict implementation of international standards by different venues such as CODEX to avoid the increase in prevalence of coagulase positive and coagulase negative *Staphylococcus* isolates and their antibiotic resistance genes in imported beef meat at the Egyptian market.

## Background

Contamination of meat with foodborne pathogens represents a major public health threat. The increasing volume of trade and travel is considered as a potential risk factor facilitating the global transport and dissemination of pathogenic bacteria in food. Imported animal products are considered as a clear example, and risk analyses have been previously applied to characterize these products [1]. This risk analyses strategy have been implemented following the World Trade Organization (WTO) creation. The application of sanitary and phytosanitary measures in response to the sanitary and phytosanitary measures agreement (SPS Agreement) [2] requires the WTO members to remove barriers on the trade of agricultural products, except in situations where such trade can potentially create risk to the animal, human or plant health in the importing country.

*Staphylococcus aureus* is one of the most common foodborne pathogens causing food poisoning outbreaks worldwide [3]. Other than *S. aureus*, the clinical and veterinary importance of coagulase-positive staphylococci (CPS) and coagulase-negative staphylococci (CNS) have often been neglected. In recent years, the risk of CPS and CNS has been highlighted by recent reports [4–6] with special reference to the CNS that have been commonly found in food [7, 8]. CNS have been recorded as conveying vector for virulence genes and have been implicated in some cases of food poisoning [9]. Furthermore, food-related staphylococci could act as dissemination vectors for antibiotic resistance genes to other potentially pathogenic microorganisms causing immediate threat to the public health. The *mecA*-harboring CNS (MRCNS) have been reported to have a reservoir in animal farm facilities and in meat products, with the ability to be conveyed to *S. aureus* [10–12].

The remarkable global concern of antibiotic- resistant pathogens in the food chain and the potential for these resistant pathogens to spread through the food chain prompted the Codex Alimentarius Commission to establish an *ad hoc* Intergovernmental Task Force on antimicrobial resistance. The main task of this commission is to apply a complete risk assessment strategy on the use of antimicrobials belonging to both clinical and veterinary classes. While domestic control over antimicrobial usage policies and monitoring is achievable, negligible information is available for imported food [13], with special reference to beef meat that has been incriminated to contribute to the emergence of multidrug resistance among humans through the dispersion of resistance genes carried by resistant pathogens transmitted by contaminated meat [14, 15].

Livestock-associated methicillin-resistant *S. aureus* (LA-MRSA) have acquired a number of novel and unusual antimicrobial resistance genes including multi-resistance genes such as the *cfr* gene, that confers resistance to phenicols, lincosamides, oxazolidinones, pleuromutilins, and streptogramin A [16]. Oxazolidinones are last resort antimicrobial agents for the control of serious infections caused by MRSA and vancomycin-resistant enterococci in humans. MRSA is also notoriously difficult to treat due to resistance to β-lactams (including penicillin, oxacillin, and methicillin) represent a class of antibiotics generally prescribed as the first line of defense against clinical infections caused by staphylococci, which include drugs like penicillin, oxacillin, and methicillin. This resistance was attributed to the carriage of *mecA*. The *mecA* gene encodes a different form of penicillin-binding protein, PBP2a, which β-lactam drugs cannot inactivate.

Therefore, the objectives of this study were to characterize the diversity of *Staphylococcus* strains recovered from imported meat sold in the Egyptian market and to assess recovered isolates as potential dispersion vectors for the spread of antimicrobial resistance. To achieve that aim, *Staphylococcus* isolates were tested for the presence of antimicrobial resistance phenotypes and genetic determinants (*mecA*, *cfr*, *gyrA*, *gyrB*, and *grlA*) and for their biofilm formation.

## Methods

### Imported beef meat samples

A total of 100 imported frozen meat samples were delivered to the Department of Poultry Diseases, Animal Health Research, Institute, Dokki, Egypt, as a routine microbiological analysis check for foodborne pathogens. Samples were collected by the food hygiene officials in ice-boxes from different supermarket chains as well as butcher shops located in the Great Cairo Zone. The meat samples (25 g) were suspended in 225 ml sterile phosphate buffered saline (PBS, pH 7.4) and homogenized in a stomacher (Lab-Blender 400, PBI, Milan, Italy) for 10 min. A total of 25 ml of the homogenate were added to 10 ml of Giolitti-Cantoni broth (BD Diagnostics, Franklin Lakes, NJ, USA). The tubes were incubated at 37 °C for 18–24 h with shaking at 200 rpm. Ten microliter aliquots of the enriched cultures were seeded on Baird Parker agar (BD Diagnostics, Franklin Lakes, NJ, USA), supplemented with egg yolk tellurite emulsion. The plates were incubated at 37 °C for 18–24 h and recovered single colonies were streaked onto blood agar plates (TSA with 5% sheep blood), and further incubated at 37 °C for 12–18 h. Characteristic staphylococci colonies (black, with or without a halo) were further identified based on Gram stain, catalase assay, tube coagulase test and further biochemical identification tests referred in standard diagnostic tables [17].

### Determination of virulence factors

#### Production of hemolysins

Production of α-, β- and γ-hemolysins was detected by streaking each staphylococcal isolate on blood agar plates containing 5% sheep red blood cells following the protocol previously developed [18].

#### Vero cell cytotoxicity assay

The ability of the isolated staphylococci to initiate degeneration of Vero cells was microscopically evaluated following the validated methodology described elsewhere [19] with minor modifications. The cytotoxicity assay was carried out using Vero (African green monkey kidney) cells in 96-well microtiter trays. Suspensions of each tested strain in distilled water were adjusted to 0.5 McFarland standard. Then, 20 μl of bacterial suspensions were added to 3.5 ml of brain heart infusion broth (BBL, Becton–Dickinson Microbiology Systems). The tubes were incubated 2 days at 35 °C, and thereafter for 2 days at room temperature. After centrifugation of bacterial broth cultures, 20 μl of supernatants were added in triplicate to 180 μl of cell culture medium. After incubation at 37 °C, in a humid atmosphere and 5% CO_2_, the cytotoxic effect of each staphylococcal strain on the Vero monolayer morphology was microscopically perceived up to 5 days.

### Phenotypic determination of the antibiotic resistance profile

The disk diffusion method was used to investigate the antibiotic resistance phenotype of the 23 *Staphylococcus* spp. recovered from the imported beef meat on Mueller–Hinton agar plates as previously described [20]. *Escherichia coli* NCIMB 50034 and *S. aureus* ATCC 25923 were included as controls. The antibiotics tested were selected from two categories, as follows [21]: (i) Critically important antibiotics: ampicillin-sulbactam (20 µg), methicillin (5 µg), oxacillin (1 µg), penicillin (10 µg), ciprofloxacin (5 µg), erythromycin (15 µg), gentamicin (10 µg), vancomycin (30 µg) and (ii) Highly important antibiotics: chloramphenicol (30 µg), clindamycin (2 µg), tetracycline (30 µg), and sulfamethoxazole/trimethoprim (25 µg).

### Molecular characterization

Overnight cultures of all *Staphylococcus* isolates were subjected to the boiling method for the preparation of cell lysates to be used for DNA templates. Genus-specific confirmation was carried out through PCR using the 16S rRNA gene *Staphylococcus*-genus-specific primers and cycling conditions previously described [22]. *S. aureus* ATCC 43300 and *E*. *coli* NCIMB 50034 were used as positive and negative controls, respectively. All confirmed staphylococci were submitted to further molecular analyses for the detection of the *mecA* gene, that is considered as the gold-standard for MRSA confirmation [23]. Four additional antimicrobial resistance markers frequently reported in *S. aureus* were screened by PCR, including the *gyrA, gyrB*, and *grlA* genes that are responsible for quinolone resistance and the *cfr* gene (chloramphenicol-florfenicol resistance gene), conferring resistance to several classes of antibiotics (phenicols, lincosamides, oxazolidinones, pleuromutilins, and streptogramin A; known as the PhLOPSA phenotype). PCR amplifications were carried out with a GeneAmp PCR System 2400 (Perkin-Elmer, Weiterstadt, Germany) using the primers and the cycling conditions previously described [8]. Two microliters of template DNA were added to 23 µl of master mix containing 1 µM of each primer and 3U of Taq polymerase. The amplicons were screened by gel-electrophoresis on 1.5% (w/v) agarose gels in TBE buffer and visualized following ethidium bromide staining. *S. aureus* reference strains EMRSA-15 and ATCC 25923 were used was used as a positive controls for the *mec* *A* and the *cfr* genes, respectively.

### Biofilm formation

The tube method (TM) and Congo red agar (CRA) method previously described [24] were followed to assess the ability for biofilm and slime formation by the 23 *Staphylococcus* isolates using the following international reference strains as controls: the non-biofilm producers *Staphylococcus epidermidis* ATCC 12228 and *Staphylococcus warneri* ATCC 10209 (negative controls), and the biofilm producers *S. epidermidis* ATCC 35983, *Staphylococcus simulans* ATCC 27851 and *Staphylococcus xylosus* ATCC 29979 (positive controls). For the TM, adherence was observed as a ring formation on the inside walls of the test tube when stained with crystal violet. Regarding the interpretation of the TM results, strains were classified as strongly adherent (+) or negative (−). Results of the CRA method were interpreted as follows: very black, black and almost black colonies were considered to be strong biofilm formers, while very red, red and bordeaux colonies were classified as negative strains for biofilm formation.

### Correlation analyses

Pearson’s correlation was used to analyze the association between all studied phenotypic and genotypic features. Univariate analyses (Chi square test, *p* < 0.05) were performed to identify variables significantly associated. The *p* values < 0.05 were considered statistically significant. Statistical analysis and data representation were done using R software (3.3.2).

## Results

### Isolation and characterization of *Staphylococcus* spp. from imported beef meat samples

In this study, a total of 23 staphylococcal isolates were recovered from 100 imported meat samples (23%), including CPS strains (12/23) and CNS strains (11/23) (Table 2). The 23 *Staphylococcus* spp. (*S. aureus,* n = 3; *S. hyicus,* n = 6; *S. intermedius,* n = 3; *S. epidermidis,* n = 1; *S. hemolyticus,* n = 1; *S. hominis,* n = 1; *S. lugdunensis,* n = 3; *S. simulans,* n = 1; and *S. scuri,* n = 4) isolates were cytotoxic to Vero cells. The three types of hemolysis were manifested as α-, β- and γ-hemolysins at a percentage of 17.4, 47.8, and 34.8%, respectively (Table 1).

**Table 1:** Phenotypic (antibiotic resistance, haemolytic activity and biofilm formation ability) and genotypic (antibiotic resistance genes) profile of all *Staphylococcus* spp. strains isolated from imported meat

Isolate	Species	Antibiotic resistance genes					Antibiotic resistance profile												Biofilm formation		Haemolysis			Virulence
		*mecA*	*gyrA*	*grlA*	*gyrB*	*Cfr*	Ampicillin-sulbactam	Chloramphenicol	Ciprofloxacin	Clindamycin	Erythromycin	Gentamycin	Methicillin	Oxacillin	Penicillin	Sulfamethoxazole/trimethoprim	Tetracycline	Vancomycin	Congo Red Agar	Tube method	α	β	δ	Vero cell
1	*S. aureus*	−	+	−	−	−	R	S	R	R	R	S	S	R	R	R	S	S	−	+	−	+	−	+
2	*S. aureus*	−	+	−	−	−	S	S	S	S	S	S	S	S	R	R	S	S	−	+	−	−	+	+
3	*S. aureus*	−	−	−	+	−	S	S	S	S	S	S	S	S	R	R	S	S	−	+	−	−	+	+
4	*S. hyicus*	+	−	+	−	−	S	S	S	R	S	R	R	R	R	R	S	R	+	+	−	+	−	+
5	*S. hyicus*	−	+	−	−	−	S	S	S	S	S	S	S	S	R	R	S	S	−	+	−	+	−	+
6	*S. hyicus*	−	−	+	−	−	S	S	S	S	S	S	S	S	R	R	S	S	−	+	−	+	−	+
7	*S. hyicus*	+	+	+	+	−	S	S	R	R	S	R	R	R	R	R	R	R	+	+	+	−	−	+
8	*S. hyicus*	+	−	−	−	−	S	S	R	R	S	R	R	R	R	R	S	R	+	+	−	−	+	+
9	*S. hyicus*	−	+	+	−	−	S	S	S	R	S	R	S	S	R	R	S	S	−	−	−	−	+	+
10	*S. intermedius*	−	+	−	−	−	S	S	S	R	R	R	S	S	R	R	S	S	−	−	−	+	−	+
11	*S. intermedius*	−	+	+	+	−	S	S	S	R	R	R	R	R	R	R	S	R	+	+	−	−	+	+
12	*S. intermedius*	+	−	+	+	−	S	S	R	R	R	R	R	R	R	R	R	R	+	+	−	−	+	+
13	*S. epidermidis*	−	+	+	−	−	S	R	R	R	R	R	S	R	R	R	S	S	−	+	−	+	−	+
14	*S. hemolyticus*	−	−	−	−	−	S	S	S	S	S	S	S	S	S	S	S	R	+	+	−	+	−	+
15	*S. hominus*	−	−	−	−	−	R	S	S	R	R	R	R	R	R	R	R	R	−	−	−	+	−	+
16	*S. lugdunensis*	+	−	−	−	−	S	S	R	R	S	R	R	R	R	R	R	R	+	−	−	+	−	+
17	*S. lugdunensis*	−	−	−	−	−	S	S	S	S	S	S	S	S	R	R	S	S	+	+	−	+	−	+
18	*S. lugdunensis*	−	+	+	+	−	S	S	S	S	S	S	S	S	R	R	S	R	−	+	−	+	−	+
19	*S. simulans*	−	+	+	−	−	S	S	R	R	S	R	S	R	R	R	R	S	−	+	−	+	−	+
20	*S. scuri*	−	+	+	+	−	R	S	R	R	S	R	R	R	R	R	R	S	+	+	−	+	−	+
21	*S. scuri*	−	+	−	−	−	R	S	S	R	S	R	S	S	R	R	S	S	−	+	−	−	−	+
22	*S. scuri*	−	−	−	−	−	R	S	S	R	S	R	S	R	R	R	S	S	−	+	−	−	−	+
23	*S. scuri*	−	−	−	−	−	R	S	S	S	S	S	S	R	R	R	S	S	−	+	−	−	−	+

### Biofilm formation phenotype

Using the tube method (TM), a total of 18 isolates (78.3%) were classified as positive for biofilm formation ability, while 5 isolates (21.7%) were considered negative. Using CRA method, 7 of the 23 *Staphylococcus* spp. isolates (30.4%) showed black colonies with shriveled lucent texture whereas 16 (69.6%) isolates showed pink colored colonies with mucoid appearance on the CRA plates that were interpreted as negative for biofilm formation (Table 1).

### Antibiotic resistance phenotypic profile

The diversity in occurrence of antibiotic resistance among the tested *Staphylococcus* spp. isolates is outlined in Tables 1 and 2 . The 23 isolates were resistant to at least one antibiotic. Less than 50% of the isolates exhibited resistance to the β-lactams ampicillin (6/23) and methicillin (8/23), erythromycin (6/23), chloramphenicol (1/23), ciprofloxacin (7/23), vancomycin (9/23), and tetracycline (6/23) (Table 2). Ninety six percent of the isolates (22/23) were resistant to penicillin and sulfamethoxazole/trimethoprim, while only one isolate was resistant to chloramphenicol (Table 1). MDR, defined as resistance to ≥3 antimicrobial classes, was observed in 16 *Staphylococcus* isolates. Fifteen multidrug resistance (MDR) combination patterns were observed (Table 2). The penicillin/sulfamethoxazole/trimethoprim (P/SXT) resistance phenotype was evident in 14/15 of these combinations.

**Table 2:** Antimicrobial resistance patterns among *Staphylococcus* spp. strains isolated from imported beef meat

Antibiotics	Number of antibiotics	Total number of isolates	%
VA	1	1/23	4
P, SXT	2	6/23	26
P, TE, VA, SXT	4	1/23	4
P, CN, E, DA, SXT	5	2/23	9
P, OX, CN, DA, SXT			
P, OX, SAM, CN, DA, SXT	6	3/23	13
P, OX, MET, CN, DA, SXT			
P, OX, E, DA, C, SXT, CIP	7	5/23	22
P, CN, DA, TE, VA, SXT, CIP			
P, OX, CN, DA, TE, SXT, CIP			
P, OX, MET, CN, DA, VA, SXT			
P, OX, MET, CN, DA, VA, SXT, CIP	8	2/23	9
P, OX, MET, CN, E, DA, TE, VA, SXT	9	3/23	13
P, OX, MET, CN, E, DA, VA, SXT, CIP			
P, OX, MET, SAM, E, DA, TE, VA, SXT			

*C* chloramphenicol, *CIP* ciprofloxacin, *CN* gentamycin, *DA* clindamycin, *E* erythromycin, *MET* methicillin, *OX* oxacillin, *P* penicilin, *SAM* ampicillin-sulbactam, *SXT* sulfamethoxazole/trimethoprim, *TE* tetracycline, *VA* vancomycin

### Antimicrobial resistance genes

From the 23 screened isolates, five isolates (3/6 *S. hyicus*; 1/3 *S. intermedius*; 1/3 *S. lugdunensis*) were identified as positive for *mecA* (5/23). Four of the 13 oxacillin-resistant isolates harbored the *mecA* gene, while 4 of the 8 methicillin resistant isolates carried the *mecA* gene (Table 3).

**Table 3:** Prevalence of the *mecA* gene in imported beef samples

*Staphylococcus* spp. (n = number of isolates)	n = of MRS	Presence of *mecA*gene		n = of MSS	Precence of *mecA* gene	
		n=	%		n=	%
*S. aureus* (n = 3)	0	0	0	3	0	0
*S. hyicus* (n = 6)	3	3	100	3	0	0
*S. intermedius* (n = 3)	2	1	50	1	0	0
*S. epidermidis* (n = 1)	0	0	0	1	0	0
*S. hemolyticus* (n = 1)	0	0	0	1	0	0
*S. hominus* (n = 1)	1	0	0	0	0	0
*S. lugdunensis* (n = 3)	1	0	0	2	1	50
*S. simulans* (n = 1)	0	0	0	1	0	0
*S. scuri* (n = 4)	1	0	0	3	0	0
Total	8	4	50	15	1	6.7

*MRS* methicillin resistant *Staphylococcus*, *MSS* methicillin susceptible *Staphylococcus*

Interestingly, the following three observations were recorded: (i) although *S. hominis* and *S. hemolyticus* isolates were resistant to methicillin and vancomycin, respectively, the resistance genes *mecA*, *gyrA*, *gyrB*, *grlA* and *cfr* were absent. (ii) three *S. aureus* isolates did not carry the *mecA* gene (100%) and were phenotypically characterized as MSS; and (iii) one *S. lugdunensis* isolate was observed to harbor the *mecA* resistance gene but was phenotypically characterized as methicillin-susceptible non-*S. aureus* (MSNSA). The resistance gene *mecA* was detected in 4/8 (50%) methicillin-resistant non-*S. aureus* (MRNSA) isolates (Table 3). Of the three MSSA isolates, two carried the *gyrA* gene (66.66%) and one carried the *gyrB* gene (33.33%) (Table 1). The *cfr* gene was absent in all *Staphylococcus* isolates. The non-*cfr*-conveying CNS showed extensive resistance to several antimicrobials irrespective of those incorporated in the *cfr*-transmitted PhLOPSA phenotype (conferring resistance to several classes of antibiotics **(**phenicols, lincosamides, oxazolidinones, pleuromutilins, and streptogramin A; PhLOPSA phenotype) (Table 1). Phenotypically, 40% (8/20) of NSA (non *Staphylococcus aureus*) isolates were methicillin resistant, while the *mecA* gene was detected in only 25% (5/20) of isolates.

### Correlation analyses

**Fig. 1:**
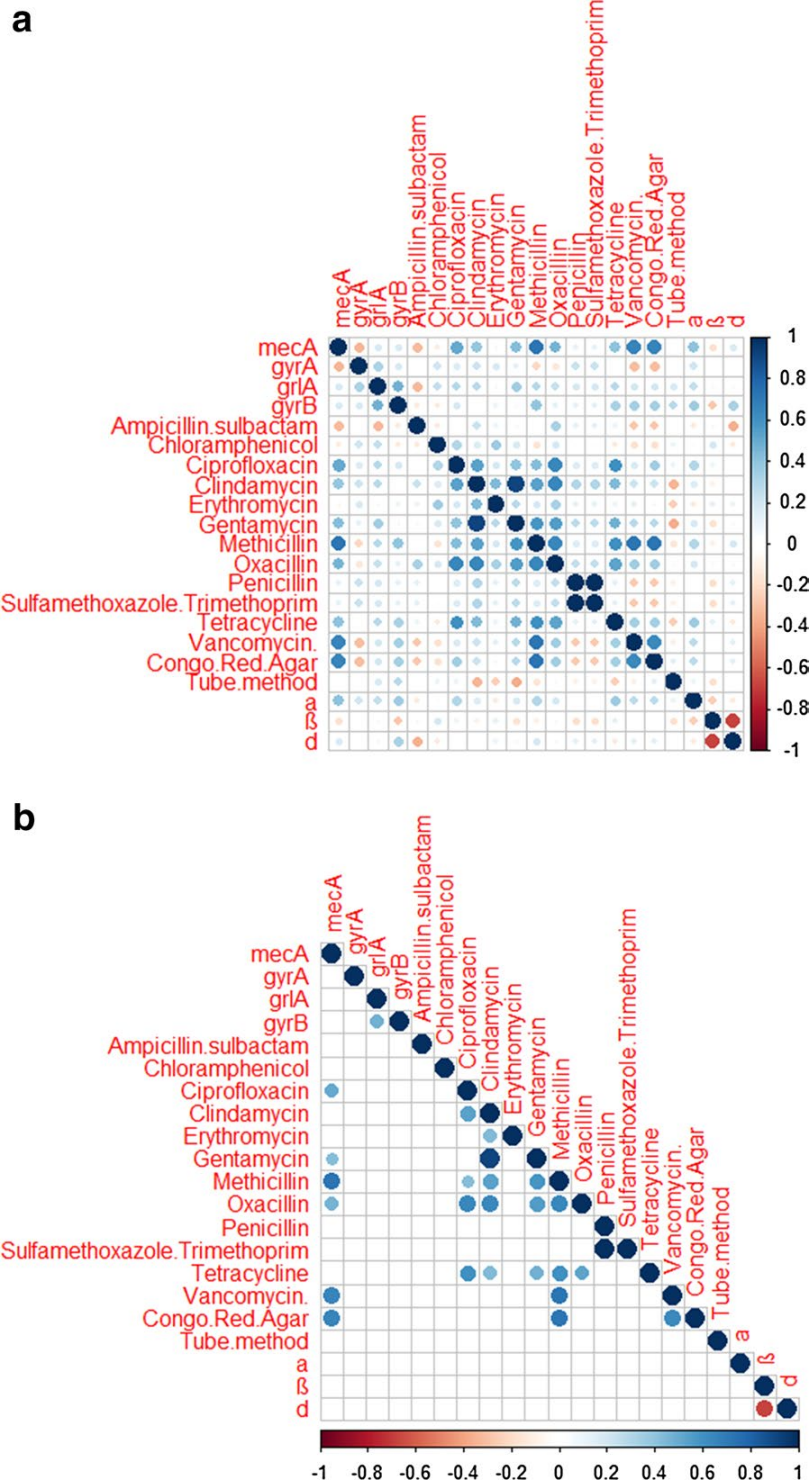
**a** Correlation matrix of phenotypic (antibiotic resistance, haemolytic activity and biofilm formation ability) and genotypic (antibiotic resistance genes) features. **b** Correlation matrix showing only significant (p < 0.05) associations, as assessed by the Chi square test

Correlation analyses showed that presence of *mecA* was directly correlated with resistance to ciprofloxacin, gentamicin, methicillin, oxacillin, and vancomycin (Pearson's correlation coefficients of 0.50, 0.42, 0.72, 0.46 and 0.66, respectively) (Fig. 1). There were several instances of co-occurrence of resistance to various antibiotics. Indeed, resistance to various antibiotics was directly correlated. For instance, resistance to methicillin was significantly associated with resistance to ciprofloxacin (Pearson's correlation coefficient of 0.43), clindamycin (Pearson's correlation coefficient of 0.53), gentamicin (Pearson's correlation coefficient of 0.59), oxacillin (Pearson's correlation coefficient of 0.64), tetracycline (Pearson's correlation coefficient of 0.61) and vancomycin (Pearson's correlation coefficient of 0.72). Similarly, a high correlation between resistances to several other antibiotics was found (Fig. 1). In addition, biofilm formation, as assessed by the CRA tests, was directly correlated with *mecA* presence (Pearson's correlation coefficient of 0.66), methicillin resistance (Pearson's correlation coefficient of 0.72) and vancomycin resistance (Pearson's correlation coefficient of 0.63).

## Discussion

Globally, an increasing recognizable concern exists on the status of antimicrobial resistant microbial contaminants in the food chain and their capacity to be widely dispersed through the international trade of food. This prompted the Codex Alimentarius Commission to establish an ad hoc Intergovernmental Task Force on Antimicrobial Resistance in 2007, bearing in mind the occurrence of national and regional diversity in antimicrobial misuse, human subjection to resistant microorganisms and determinants and their prevalence in foodborne pathogens. Egypt imports different meat types to fill the gaps in animal protein supply. According to the U.S. Meat Export Federation (USMEF) that examines key statistics and trends in beef and pork trade from 2008 to 2017, Egypt’s projection for beef-importing in 2017 would reach about 332,000 metric tons of meat imports and this is supposed to grow to 2025 by 52.28% [25]. While the importation of beef meat in Egypt is crucial to close the gap in animal protein requirements, monitoring the frequency of antimicrobial resistance in imported meat must assure that quality and safety standards are met.

The spread of MRSA is a serious public health concern for both human and veterinary medicine. Due to the occurrence of MRSA, methicillin and other β-lactamic antibiotics have become useless for clinical therapy, leaving the term MRSA to be used to describe *S. aureus* strains resistant to effectively all β-lactamase-resistant penicillins and harboring the *mecA* gene [23]. Although the development and spread of multiple antibiotic-resistant MRSA have gained much attention over the past years, yet the role of imported meat has not been given much attention. In a Danish study, Agersø et al. reported that MRSA was found in 18% of the imported broiler meat and 7.5% of the imported pork [26]. MRSA are often resistant to other antimicrobials different to methicillin, highlighting the necessity for new and effective antimicrobials. Attributed to their extended antimicrobial spectra, fluoroquinolone compounds such as ciprofloxacin and norfloxacin were recommended as useful candidates for eradicating MRSA [27]. Nonetheless, due to the misuse of these compounds in the clinical practice, resistance of MRSA to these compounds has been observed [28]. For this reason ciprofloxacin is not recommended to be used in empirical therapy against MRSA infections. Furthermore, the use of other fluoroquinolones is only allowed following accurate antimicrobial susceptibility testing. Nonetheless, vancomycin remains the drug of choice to treat MRSA infections [29].

Identification of methicillin resistant staphylococci in the laboratory is often problematic due to difficulty in detecting heterogeneous methicillin-oxacillin resistance in staphylococci [30, 31]. Moreover, Standard interpretive breakpoints for oxacillin susceptibility reporting published by the CLSI (formerly the National Committee for Clinical and Laboratory Standards) were changed in 2004, and NSA isolates of veterinary origin are now more likely than in previous years to be deemed resistant by testing laboratories that use those guidelines [32].

In this study, the susceptibility of 23 *Staphylococcus* spp. strains isolated from imported meat to 12 antibiotics from different classes was evaluated. An extreme resistance was found against penicillin and sulfamethoxazole/trimethoprim as compared to other tested antibiotics, which could be attributed to the extensive use of these antibiotics in treating mastitis cases and/or as growth promoters in animal feed in the countries from which the beef meat was imported. Resistance to other clinically important antibiotics, including β-lactamic antibiotics (such as ampicillin-sulbactam, methicillin, oxacillin), fluoroquinolones (ciprofloxacin), macrolides (erythromycin), aminoglycosides (gentamicin), glycopeptides (vancomycin), lincosamides (clindamycin) and tetracycline also occurred and, in some cases, multidrug resistance phenotypes were identified. Indeed, correlation analyses showed co-occurrence of resistance to series of antibiotics. Extended-spectrum beta-lactamases (ESBL) -producing pathogens are often resistant to fluoroquinolones and aminoglycosides since resistance mechanisms for these classes of antibiotics are often carried on the same large plasmids that contain the genetic elements for ESBL production [33]. Our results corroborate this observation. Thus, for instance, resistance to methicillin was significantly associated with resistance to ciprofloxacin and gentamycin, among others (clindamycin, oxacillin, tetracycline and vancomycin). The colonization ability in conjunction with the occurrence of MDR highlights the hazardous nature of the *Staphylococcus* spp. isolated from imported beef meat.

The antimicrobial resistance determinants *mecA*, *gyrA*, *gyrB*, *grlA* and *cfr*, frequently reported in *S. aureus* strains were also screened in recovered isolates. The emergence of the multiple drug resistance gene *cfr* in staphylococci is of global clinical and veterinary importance that has been previously investigated in staphylococci [34] and has been identified as a phenicol and lincosamide resistance gene [35]. In this study, the absence of the *cfr* gene among imported beef meat isolates suggests a decreased potential risk as an outcome of harboring the gene in the food chain through imported meat. Furthermore, the absence of the gene *cfr* poses a significant and interdisciplinary public health positive factor to the Egyptian consumers. One isolate showed chloramphenicol resistance but was negative for the *cfr* gene. This observation might be attributed to a possible heterogeneous expression of the *cfr* gene [36] or to different potential chloramphenicol resistance mechanisms.

The *mecA* gene confers methicillin resistance. It encodes the penicillin binding protein 2a, an enzyme that has low affinity for beta-lactams, and has been reported to lead to resistance to ciprofloxacin, gentamycin, methicillin, oxacillin and vancomycin [37]. In our study, the *mecA* gene was carried by 5 out of 23 *Staphylococcus* spp. isolates, and, as expected, presence of *mecA* was directly correlated with resistance to ciprofloxacin, gentamycin, methicillin, oxacillin, and vancomycin. Nevertheless, not all methicillin or oxacillin resistant isolates were *mecA* positive. It was previously indicated that some isolates missing the *mecA* gene could be identified as phenotypically resistant to oxacillin (MRSA) [38]. These results suggests that other potential resistance mechanisms might exist. Molecular investigations of a *S. aureus* isolate, which was found to be phenotypically resistant to methicillin but negative for the *mecA* gene, were able to identify the presence of a novel *mecA* homologue, which was found to be associated with cattle [39], suggesting the existence of a zoonotic MRSA reservoir [40]. In 2012, the International Working Group on the Classification of Staphylococcal Cassette Chromosome elements (IWCC) renamed the *mecA* variant, *mecC* [41]. Methicillin-resistant *S. aureus* strains carrying the *mecC* gene have been shown to cause a range of infections in humans and appear to be predominantly community associated [40]. The prevalence of *mecC* in CNS has been recently reported for 13 European countries, where it has been isolated from 14 different host species (Holmes et al. 42), and an allotype of the *mecC* gene has been detected in a *S. xylosus* strain [42]. It is also worth mentioning the fact that one *S. lugdunensis* isolate was observed to harbor the *mecA* resistance gene but was phenotypically characterized as MSNSA. The phenotypic susceptibility to oxacillin, methicillin, penicillin and ampicillin (β-lactam antibiotic of the penicillin class) regardless of the *mecA* presence may be attributed to the heterogeneous expression of *mecA* [43], which is more common in CNS than that in *S. aureus* [44]. Nevertheless, it should be noted that in 2014, Bhargava and Zhang emphasized the importance of MRCNSs as important reservoirs of *mecA* that could act as precursors of MRSA [11]. In addition, temperature abuse during storage and transportation to the importing country may result in multiplication of MRS [45]. An additional important finding was introduced by the identification and characterization of another gene on the SCC element called *mecR2* that regulates and increases *mecA* expression when MRSA bacteria encounter β-lactam drugs [46].

Besides resistance genes, mutation-mediated resistance is especially common among resistances to synthetic antibacterial agents, such as fluoroquinolones and oxazolidinones in *S. aureus* and other bacterial species [47]. The primers used in this study amplified only the copies of *gyrA, gyrB*, and *grlA* that contained mutations in the quinolone resistance-determining regions (QRDRs). In this study, the amplification of a *grlA* and *gyrA* product represented the presence of a mutation conferring resistance. Mutations in the *gyrB* gene does not play an important role in quinolone resistance [48]. Although the presence of resistance genes are a primary reason for antibiotic resistance, the resistance phenotype and gene presence are not exclusively linked in this study. Similarly, this inconsistency was common in other classes of antibiotics, demonstrating that the presence of a certain resistance gene was not necessary an indicator of antibiotic resistance [49]. A potential explanation for this inconsistency could be the lack of expression of some of the resistance genes and the presence of other genes encoding resistance. In addition, chromosomally encoded multidrug resistance pumps have been shown to have other primary functional or structural roles [50].

The connection between microbial biofilms and antibiotic resistance is of considerable interest to biomedical researchers. Previous reports have suggested that a correlation between antibiotic resistance and biofilm formation ability exists, a phenomenon that may be responsible for a decrease in the efficaciousness of antimicrobial agents against *S. aureus* infections [51]. As a consequence of biofilm development, the capability of horizontal gene transfer is increased and may facilitate the dispersion of antibiotic resistance [52]. In the present study, MRS and MDR isolates showed a good potential to form biofilms. In fact, correlation analyses showed that biofilm formation, as assessed by the CRA tests, was directly correlated with *mecA* presence, methicillin resistance and vancomycin resistance, and biofilm producing MRNSA showed high resistance to almost all antibiotic classes compared to non-producers, which corroborates previous observations [53].

## Conclusions

Despite the alert generated by the outbreaks of foot and mouth disease and swine fever, which have been attributed to international trade of meat, little information or actionable risk management information is available yet on the dissemination of microbial hazards through retail imported meat. This study reveals that imported meat can act as a transmission vector for MRSA or MRNSA harboring the *mecA* gene, which may represent a risk for both human and veterinary medicine. The data obtained on the resistance of *Staphylococcus* spp. to antimicrobials, may be used for implementing an antimicrobial resistance spread monitoring and prevention program.

## Abbreviations

ESBL: extended-spectrum beta-lactamases
CNS: coagulase-negative staphylococci
CPS: coagulase-positive staphylococci (CPS)
LA-MRSA: livestock-associated methicillin-resistant *Staphylococcus aureus*
MDR: multiple drug resistance
MRNSA: methicillin-resistant non-*Staphylococcus aureus*
MRSA: methicillin-resistant *Staphylococcus aureus*
MSNSA: methicillin-susceptible non-*Staphylococcus aureus*
NSA: non *Staphylococcus aureus*
QRDRs: quinolone resistance-determining regions
